# The antidiabetic drug acarbose suppresses age-related lesions in C57BL/6 mice in an organ dependent manner

**DOI:** 10.31491/apt.2021.06.060

**Published:** 2021-06-29

**Authors:** Sneh Gupta, Zhou Jiang, Warren Ladiges

**Affiliations:** a Department of Comparative Medicine, School of Medicine, University of Washington, Seattle, WA 98195, USA

**Keywords:** Age-related lesions, acarbose, aging intervention, geropathology

## Abstract

Acarbose (Acb) is an antidiabetic drug used to reduce blood glucose by inhibiting the conversion of complex carbohydrates into simple sugars. It has also shown promise as an anti-aging drug by increasing lifespan in mice but studies have not been reported on the effects of short-term treatment in aging mice. To address this question, 20-month-old C57BL/6 male and female mice were given a standard diet, or a diet supplemented with 1000 ppm Acb for 3 months. After this period, mice were assessed for age-related lesions as readouts for the delay in the progression of aging. Results showed there was a significant decrease in lesions of the heart and kidney in mice treated with Acb suggesting that Acb can suppress cardiac and renal pathology associated with increasing age.

Aging is a complex process and affects many organs in the body, so multiple pathways need to be targeted to combat or slow the effects of aging. Acarbose (Acb) is a well-known anti-diabetic drug for type 2 diabetic patients. It has been shown to decrease plasma glucose levels and cholesterol levels by the reversible inhibition of membrane-bound intestinal alpha-glucosidase and pancreatic alpha-amylase, two enzymes needed to digest complex carbohydrates [1]. Recently, Acb has been shown to increase median and maximum life-span in mice, when treated starting at 4 months of age [2] or starting at 8 months of age [3], with a more pronounced effect in males than females. Aging increases the risk factor for cardiovascular diseases [4], kidney impairment [5], lung diseases [6] and liver diseases [7]. It was therefore of interest to see if Acb might reduce age-related damage to organs in aging mice.

Male and female C57BL/6 mice were used, 12 in each cohort. The experimental group was fed Acb (Cayman Chemical Co., Ann Arbor, MI) at 1000 ppm in their diet (prepared by TestDiet, Inc, a division of Purina Mills, Richmond, IN), while the control group was fed an identical diet without medication for 3 months starting at 20 months of age. After this period, mice were euthanized and tissues collected and slides of heart, lungs, liver and kidney were read and scored for age-related lesions using the geropathology grading platform as described [8]. The results indicate that age-related lesions in the heart (Figure 1A) and kidney (Figure 1B) were significantly less in the Acb treated group compared to the control group for both males and females. There was no significantdifference in lesion severity in the liver (Figure 1C) or lungs (Figure 1D) between treated and control mice. These observations suggest that Acb can potentially be used to decrease the risk of cardiac and renal lesions associated with aging. Type 2 diabetes is associated with kidney and heart failure [9]. Decreasing risk of heart and kidney disease by Acb is consistent with this observation. Hence, Acb could potentially be used to decrease the risk of renal and cardiac diseases for people with a family history of these conditions. Further studies would be of interest to see the effects of acarbose in combination with other drags like rapamycin, which has been shown to decrease pathological lesions in the lungs and liver, as well as the kidneys, in aging mice [10].

## Figures and Tables

**Figure 1.**
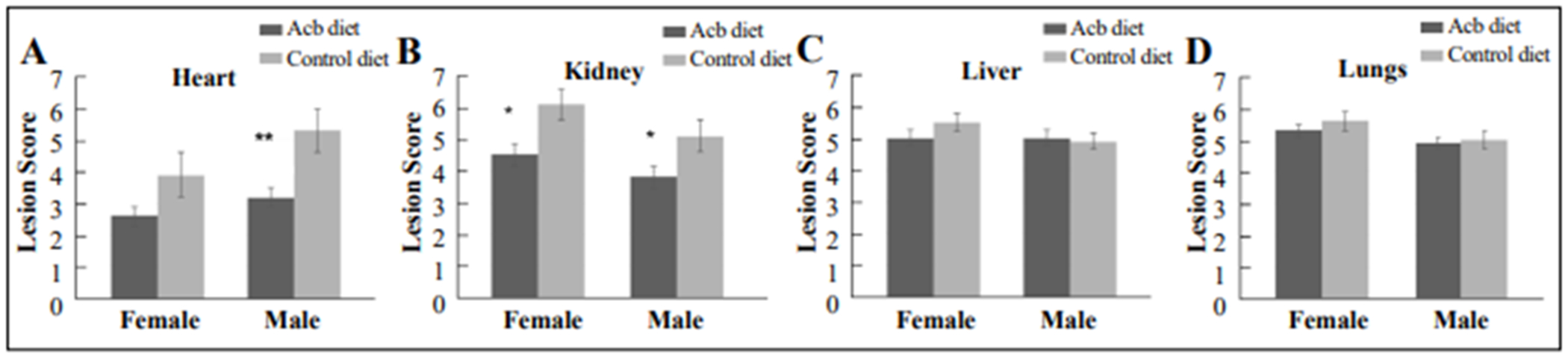
Male and female 57BL/6 mice, 20 months of age, were fed a diet containing 1000 PPM acarbose or a control diet without acarbose for three months. Tissues were collected and scored for age-related lesions. Mice fed the acarbose diet had decreased lesion scores in the heart and kidney compared to mice fed the control diet **(A and B)**, but no differences were seen in the liver or lungs **(C and D)**. * *P* ≤ 0.05.
